# Effects of storage and toothbrush simulation on color, gloss, and roughness of CAD/CAM, hand-cast, thermoforming, and 3D-printed splint materials

**DOI:** 10.1007/s00784-022-04391-3

**Published:** 2022-02-04

**Authors:** Verena Hickl, Thomas Strasser, Alois Schmid, Martin Rosentritt

**Affiliations:** Department of Prosthetic Dentistry, UKR University Hospital Regensburg, 93042 Regensburg, Germany

**Keywords:** Storage, Toothbrushing, Splints

## Abstract

**Objectives:**

The aim was to investigate color, gloss, or roughness of splint materials after storage in liquids and toothbrush simulation.

**Materials and methods:**

A total of 58 × 8 (*n* = 10 per material and group) specimens were fabricated (hand-cast, thermoforming, CAD/CAM-milled, 3D-printed materials); stored in air, water, coffee, red wine, and cleaning tablets; and investigated after fabrication, 24 h, two-, and four-week storage or toothbrushing. Color values (L*, a*, b*; ISO 11664–4:2008; CM–3500d, Konica-Minolta), gloss (ISO 2813:2014), and roughness values were determined (3D laser-scanning-microscope, KJ 3D, Keyence) before and after simulation or storage. Statistics: Levene-test, one-way ANOVA, Bonferroni post hoc test, between-subjects effects, Pearson correlation (*α* = 0.05).

**Results:**

Color, gloss, and roughness altered due to contact with staining solutions/toothbrush simulation. Highest impact on color, gloss, and roughness presented the material followed by storage time (ΔE material (η2 = 0.239/*p* < 0.001), storage time (η2 = 0.179/*p* < 0.001); gloss (η2 = 0.751/*p* < 0.001) (η2 = 0.401/*p* < 0.001); Ra/Rz (η2 ≥ 0.801/*p* < 0.001) (η2 ≥ 0.416/*p* < 0.001)). Correlations were found between Rz and Ra (Pearson 0.887/*p* ≤ 0.001) or Rz and ΔE (0.517/*p* ≤ 0.001) or Ra and ΔE (0.460/*p* ≤ 0.001).

**Conclusions:**

Storage and toothbrushing were accompanied by a change in color, gloss, and roughness. Almost all materials showed visible discoloration after 4 weeks of storage. Gloss values decreased as storage time increased. The initial roughness and polishability were better with harder materials.

Clinical relevance.

Milled and 3D printed splints show good color, gloss, and roughness resistance after 4-week storage or toothbrush application.

## Introduction

Splints are an effective therapeutic treatment of temporomandibular disorders (TMD) [1]. The appliances improve individual symptoms such as pain and functional limitations [2–4]. It is state of the art to fabricate splints on gypsum models either by applying methacrylate in the sprinkle-on technique or by vacuum thermoforming [5]. Both techniques can be combined by adjusting the occlusal surface of a thermoformed splint with acrylic resin [6, 7]. The computer-aided design/computer-aided manufacturing (CAD/CAM) opened up new possibilities for splint production [8]. The clinical situation is either recorded directly with an intraoral scanner or impressions/plaster models are scanned [9]. Based on this digital impression, the occlusal devices can be designed with a CAD software [10]. In the subtractive process, the splints are milled from a prefabricated resin-based blank using a computerized numerical control (CNC) machine [11]. A more recent approach is 3D printing with stereolithography (SLA) or digital light processing (DLP) technology [12, 13]. Here, the splints are built up and cured layer by layer by a liquid photopolymer. The mechanical properties are affected by the type of material and the processing. Post-polymerization has an important role to play in ensuring the properties of the material [14, 15]. The success of a splint treatment depends to a large extent on the patient’s compliance. Therefore, in addition to the mechanical requirements, splints should also meet esthetic [16, 17], phonetic, and functional [18] demands. Basic esthetic requirements include color stability and surface gloss [19]. Color changes can be caused by intrinsic and extrinsic factors [20] and are therefore influenced by the chemical structure and the surface of the splint. Many studies have shown that the contact of resins with various staining liquids such as coffee, red wine, and mouthwashes leads to color changes [21–23]. Color changes of ΔE < 3.3 were generally considered as a threshold and classified as clinical acceptable [24]. The gloss of the splint depends on the surface roughness and the polish. The American Dental Association (ADA) considers gloss values between 40 and 60 to be desirable [25]. Alternative literature sources define gloss values $\le$ 60 as poor finish and values between 70 and 80 as good [26–28]. Thus, the surface roughness plays an important role in the color behavior and gloss of the resins [29, 30], as well as in the accumulation of plaque and discoloring particles [31, 32]. Roughening caused by toothbrush can alter gloss and affect color stability [31, 33, 34].

The aim of the study was to investigate the effects of storage in different coloring liquids (water, coffee, red wine, and denture cleaner solution) and toothbrush simulation on color, gloss, or surface roughness of splint materials. It is expected that color, gloss, and surface roughness would change due to enduring contact with the staining solutions and toothbrush simulation. The null hypothesis was that the changes would depend on the material/fabrication, type of storage, and on the duration.

## Materials and methods

A total of 58 × 8 (*n* = 10 per material and group) specimens (diameter 10 mm, thickness 2 mm) were fabricated from hand-cast, thermoforming, CAD/CAM-milled, and 3D-printed materials (Tables 1 and 2). Hand-cast specimens (Palapress vario transparent, Kulzer, Hanau, Germany, mixing ratio 10 g powder, 7 ml liquid) were poured in silicon (VPS Hydro Putty, Henry Schein, Langen, Germany) mold and polymerized in a pressure pot (55 °C and 2 bar). Thermoforming of clear foils (Erkodur, 2.00 mm, ∅ 120 mm; Erkodent, Pfalzgrafenweiler, Germany) was performed with Erkoform-3D Motion (Erkodent, Pfalzgrafenweiler, Germany). Specimens were milled from PMMA blanks (Optimill crystal clear; Dentona, Dortmund, Germany) with Zenotec select ion (Wieland Dental + Technik, Pforzheim, Germany). 3D printing job was created with the slicing software (Netfabb, Autodesk, San Rafael, USA; print direction: 90° to the building platform; support structures were used; layer thickness 50 µm). The materials LuxaPrint Ortho Plus (DMG, Hamburg, Germany) and KeySplint Soft (Keystone Industries, Gibbstown, NY, USA) were processed with the printer “P30 + ” (Straumann, Cares P series, Basel, Switzerland). Post-processing consisted of an automated wash cycle (P Wash, Straumann, Cares P series Basel, Switzerland) and LED post-polymerization (P Cure, Straumann, Cares P series Basel, Switzerland). The materials V-Print splint and Splint Flex (Voco, Cuxhaven, Germany) were printed with Solflex 650 (Voco, Cuxhaven, Germany). Specimens were manually cleaned (2 min isopropanol bath and ultrasonic) and post-polymerized with xenon light (Otoflash G171: 2000 flashes, 2 min cooling, 2000 flashes; NK Optik, Baierbrunn, Germany). All supports and protrusions were removed with burrs and sandpaper. Polishing was performed with a finishing buff and polishing paste (Polishing unit: WP-Ex 2000 II; Wassermann, Hamburg, Germany). Finally, the discs were cleaned in an ultrasonic bath (35 °C, 10 min, Sonorex super RK 102 H, Bandelin electronic, Berlin, Germany).

**Table 1:** Materials and fabrication

System	Material	Device	LOT	Processing
Thermoforming foil	Erkodur, 2.00 mm, 120 mm^1^ *(Erkodent, Pfalzgrafenweiler, Germany)*	Erkoform-3D Motion *(Erkodent, Pfalzgrafenweiler, Germany)*	111,888/11307	/
Cast system MA	Palapress vario transparent^2^ *(Kulzer, Hanau, Germany)*	Hand-cast	K010201/K010089	Pressure pot (55°, 2 bar, 15 min)
CAD/CAM	Optimill crystal clear^3^ *(Dentona, Dortmund, Germany)*	Zenotec select ion *(Wieland Dental* + *Technik, Pforzheim, Germany)*	20,040	/
Print	LuxaPrint Ortho Plus^4^ *(DMG, Hamburg, Germany)*	P30 + *(Straumann Cares, Basel, Switzerland)*	170,211	**Printing:** Direction: 90° to building platform; layer: 50 µm **Cleaning:** P wash (Straumann Cares, Basel, Switzerland), isopropanol **Polymerization**: P cure (Straumann Cares, Basel, Switzerland), LED
Print	KeySplint Soft^5^ *(Keystone Industries, Gibbstown, NY, USA)*	P30 + *(Straumann Cares, Basel, Switzerland)*	K84189	**Printing:** Direction: 90° to building platform; layer: 50 µm **Cleaning:** P wash (Straumann Cares, Basel, Switzerland), isopropanol **Polymerization:** P cure (Straumann Cares, Basel, Switzerland), LED
Print	V-Print splint^6^ *(Voco, Cuxhaven, Germany)*	Solflex 650 *(Voco, Cuxhaven, Germany)*	2,006,565	**Printing:** Direction: 90° to building platform; layer: 50 µm **Cleaning:** Ultrasonic (2 min), isopropanol **Polymerization:** OtoFlash G171, Xenon Light: 2* 2000 flashes
Print	Splint Flex^7^ *(Voco, Cuxhaven, Germany)*	Solflex 650 *(Voco, Cuxhaven, Germany)*	V87146	**Printing:** Direction: 90° to building platform; layer: 50 µm **Cleaning:** Ultrasonic (2 min), isopropanol **Polymerization:** OtoFlash G171, Xenon Light: 2* 2000 flashes

**Table 2:** Materials and composition

Material and manufacturer	Composition
Erkodur, 2.00 mm, 120 mm *(Erkodent, Pfalzgrafenweiler, Germany)*	Thermoplastic material: polyethylenterephtalate PET-G
Palapress vario transparent *(Kulzer, Hanau, Germany)*	Methylmethacrylate-copolymer, methylmethacrylate, dimethacrylate
Optimill crystal clear *(Dentona, Dortmund, Germany)*	Methylmethacrylat, dibenzoylperoxid, methyl 2-methylprop-2-enoat
LuxaPrint Ortho Plus *(DMG, Hamburg, Germany)*	Dimethacrylate, EBPADMA, diphenyl(2,4,6-trimethylbenzoyl)phosphinoxid
KeySplint Soft *(Keystone Industries, Gibbstown, NY, USA)*	Methacrylate
V-Print splint *(Voco, Cuxhaven, Germany)*	Polyesterdimethacrylat, BIS-EMA, triethylenglycoldimethacrylat, hydroxypropylmethacrylat, diphenyl(2,4,6-trimethylbenzoyl)phosphinoxid, BHT
Splint Flex *(Voco, Cuxhaven, Germany)*	Dimethacrylat, BIS-EMA, triethylenglycoldimethacrylat (experimental test-material)

**Fig. 1:**
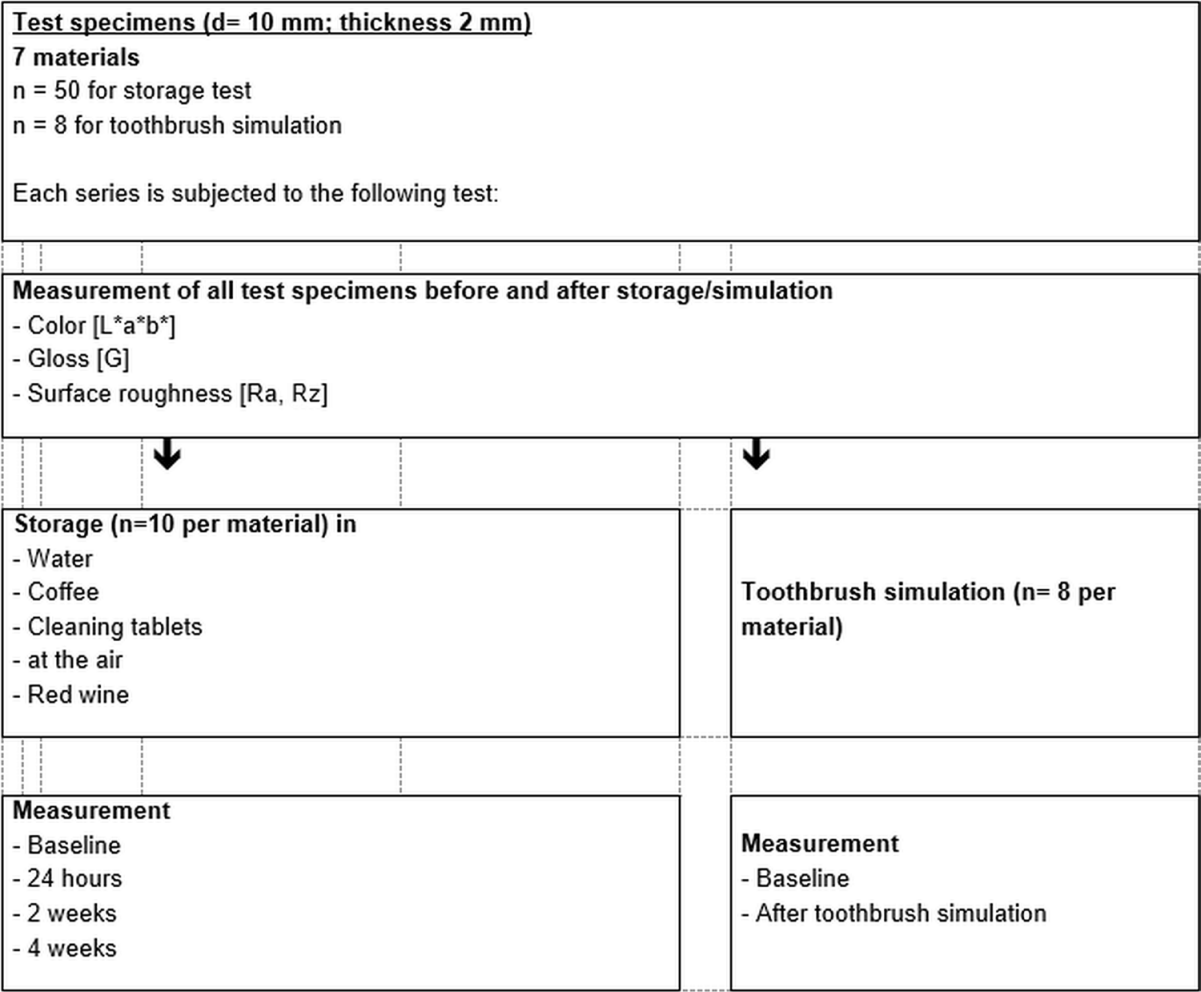
Study design

Specimens were stored in water (demineralized water), coffee (Cafet, Netto, Germany – instant coffee mild), red wine (Red wine sweet, Vino d’Italia, Italy), and cleaning tablets (Kukident – active plus, Kukident, Germany) in microwell plates. Solvents were renewed during the immersion test every 4 days. One disc was stored in 1 ml of test liquid. After storage, specimens were rinsed with water and carefully cleaned with a microfiber cloth. Specimens were investigated straight after fabrication (baseline), after 24 h, two-, and four-week storage. Specimens that were stored in air served as reference. Tooth brushing was performed with a toothbrush simulator (ZM-3; SD Mechatronik, Feldkirchen-Westerham, Germany; brush, Oral-B 1–2-3 indicator medium (35 mm), Oral B, Germany; slurry, 250 g toothpaste in 1 l demineralized water; load, 250 g, circular 10 mm movement, v = 40 mm/s, 72,000 cycles) on 8 specimens per material. Color, gloss, and roughness values were determined before and after the simulation (Fig. 1).

Color measurements were carried out using a spectrophotometer (CM–3500d, Konica-Minolta, Chiyoda, Japan) with black background. The measured color values (L*, a*, b*) were evaluated using the CIELAB system according to ISO 11664–4:2008 [35]. Color changes (ΔE) were calculated. A gloss meter (ZGM, Zehntner Testing, Sissach, Switzerland) was used to measure gloss (G) before and after immersion (angle 60°) according to ISO 2813:2014 [36]. Non-contact optical roughness values ((ISO 4287); Ra, Rz) were determined using a confocal 3D laser-scanning-microscope (KJ 3D, Keyence, Osaka, Japan; scanning area, 2400 × 1800 µm, λC = 0.8 mm). The arithmetic roughness Ra is the average of the absolute values along the single measuring section. The maximum height of the profile (maximum roughness; Rz) describes the absolute vertical distance between the maximum profile peak height and the maximum profile.

Calculations and statistical analysis were performed using SPSS 25.0 for Windows (IBM, Armonk, NY, USA). Homogeneity of the data was controlled with Shapiro–Wilk test. Means and standard deviations were calculated and analyzed using one-way analysis of variance and the Bonferroni test post hoc analysis. Between-subjects effects were investigated. The level of significance was set to *α* = 0.05. Pearson correlation between the individual parameters was determined.

## Results

### ΔE changes

**Fig. 2:**
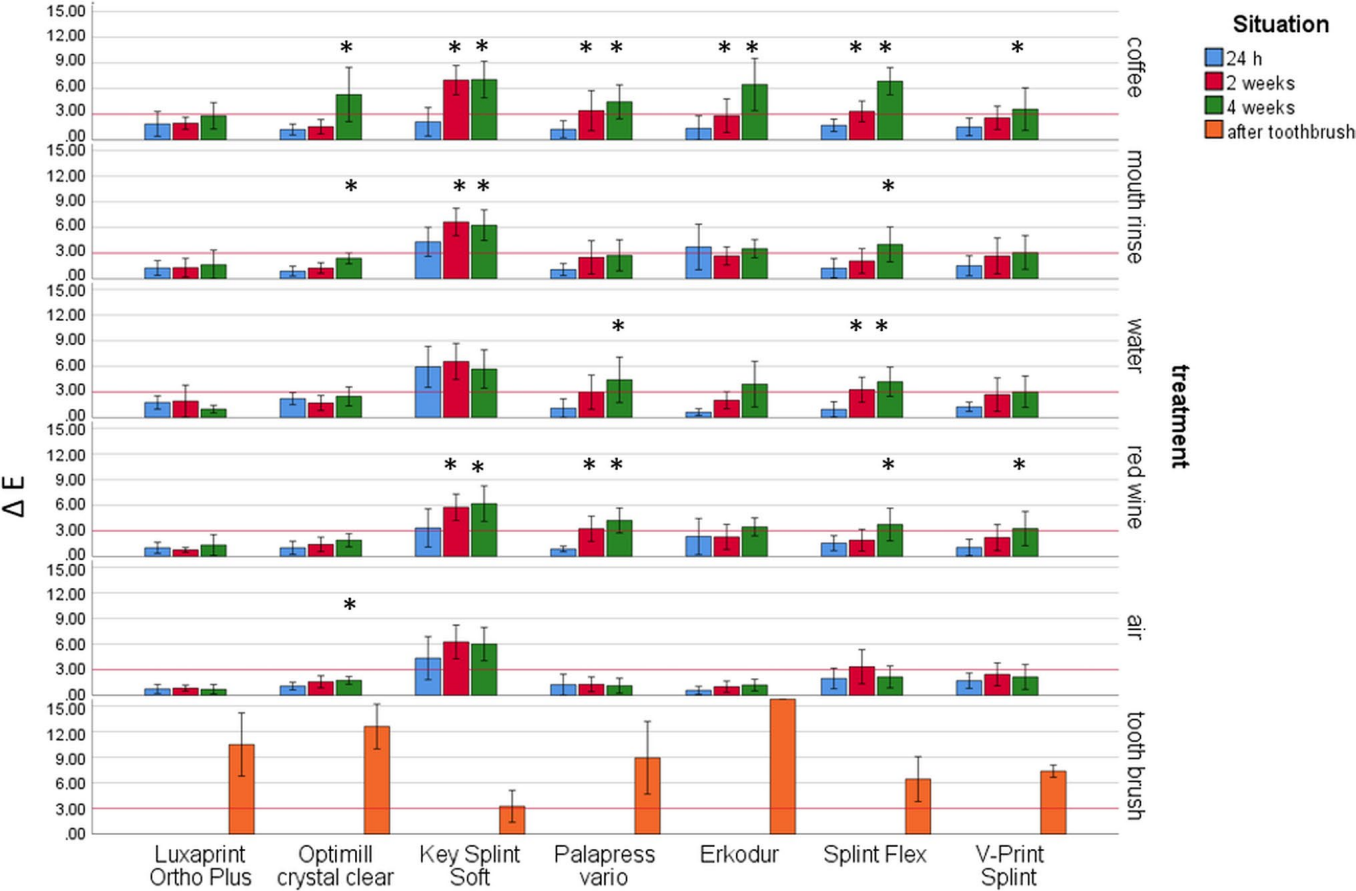
Color change ΔE after different aging/storage treatments and storage times (mean and standard deviation, * significant differences to 24 h measurement, *α* = 0.05)

Highest impact on ΔE presented the material (η^2^ = 0.239/*p* < 0.001), followed by storage time (η^2^ = 0.179/*p* < 0.001) and storage conditions and all other combinations (0.059 ≤ η^2^ ≤ 0.074/*p* < 0.001) (Fig. 2).

LuxaPrint Ortho Plus showed ΔE values between 0.69 (4 weeks air) and 2.82 (4 weeks coffee). No significant differences were found after different storage times (Anova, *p* ≥ 0.190; Bonferroni, *p* ≥ 0.329). Optimill crystal clear provided significant general differences (Anova *p* ≤ 0.028) and individual differences (*p* ≤ 0.031) after 4-week storage for coffee, mouth rinse, and air storage. ΔE varied between 0.88 (24 h mouth rinse) and 5.31 (4 weeks coffee). KeySplint Soft showed ΔE results between 2.11 (24 h coffee) and 7.06 (4 weeks coffee). Significant general differences (Anova *p* ≤ 0.011) and individual differences (*p* ≤ 0.032) after 2 weeks storage for coffee, mouth rinse, and red wine storage could be determined. Palapress vario provided significant general differences (Anova *p* ≤ 0.004) and individual differences (*p* ≤ 0.043) after 2 weeks storage for coffee and red wine as well as 4 weeks in water (*p* = 0.003). ΔE values between 0.87 (24 h red wine) and 4.45 (4 weeks coffee) were found. Erkodur showed ΔE results between 0.57 (24 h air) and 6.49 (4 weeks coffee). Significant general differences (Anova *p* ≤ 0.001) and individual differences (*p* < 0.001) after 4 weeks storage for coffee and in water (*p* < 0.001) could be determined. ΔE values for Splint Flex were between 0.97 (24 h water) and 6.87 (4 weeks coffee). Significant general differences (Anova *p* ≤ 0.014) and individual differences (*p* ≤ 0.038) were found after 4 weeks storage for coffee and red wine. V-Print splint provided no significant general differences (Anova *p* = 0.117) and individual differences (*p* < 0.001) only in air. Changes were significant after 2 weeks for storage in coffee and in water (*p* ≤ 0.019) and after 4 weeks (*p* ≤ 0.006). ΔE results between 1.04 (24 h red wine) and 3.60 (4 weeks coffee) were found.

Toothbrush abrasion provided ΔE values between 3.26 (KeySplint soft) and 20.95 (Erkodur) with significant differences between the groups (Anova *p* ≤ 0.001). Individual differences after toothbrush abrasion were found for LuxaPrint Ortho Plus and KeySplint Soft or Erkodur (*p* ≤ 0.001); Optimill crystal clear and KeySplint Soft, Erkodur, Splint Flex, or V-Print splint (*p* ≤ 0.010); KeySplint Soft and Palapress vario or Erkodur (*p* ≤ 0.004); Palapress vario and Erkodur (*p* = 0.000); and Erkodur and Splint Flex or V-Print splint (*p* ≤ 0.001).

### Gloss

**Fig. 3:**
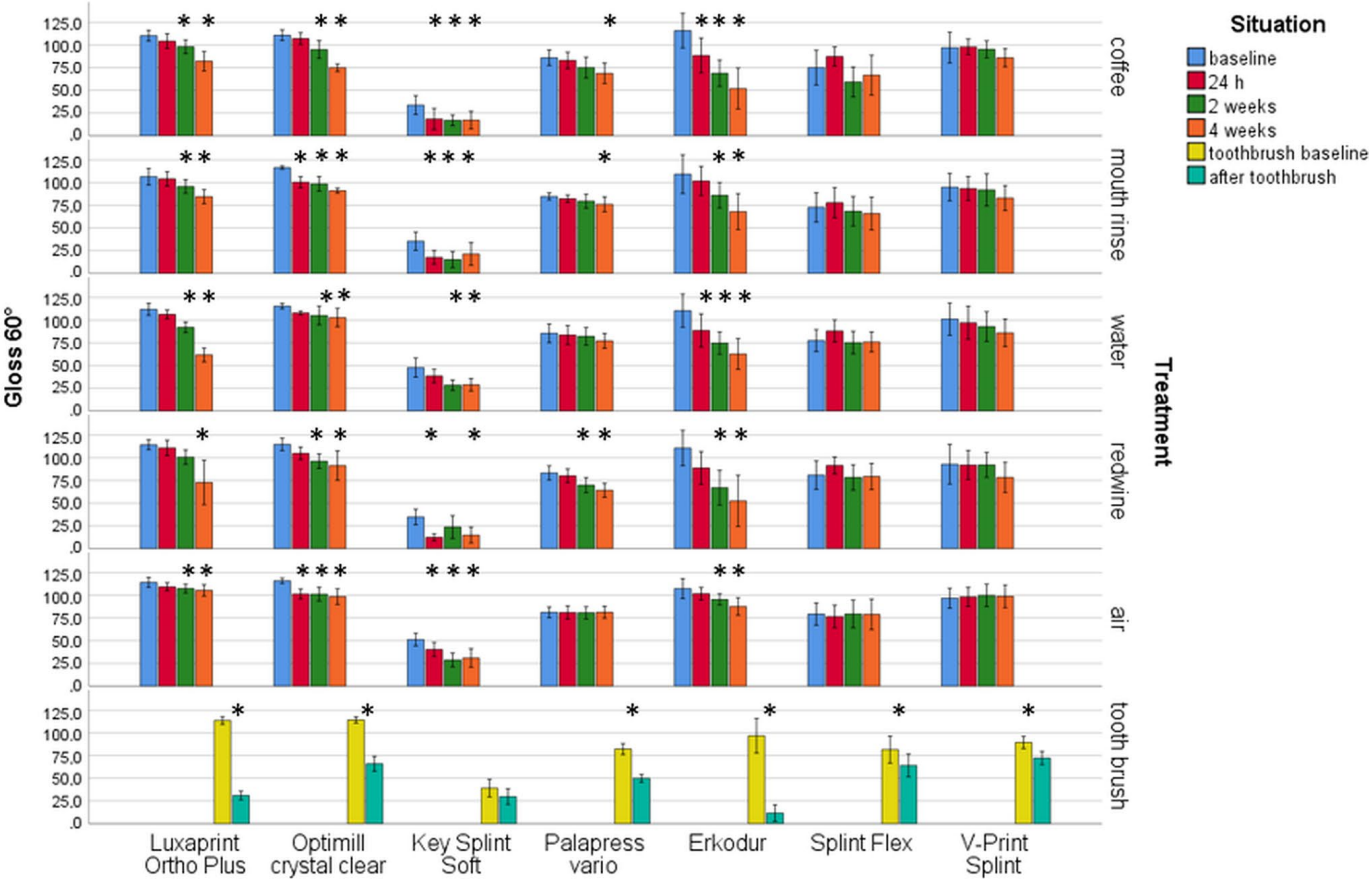
Gloss (60°) after different aging/storage treatments and storage times (mean and standard deviation, * significant differences to baseline measurement, *α* = 0.05)

Highest impact on gloss presented the material (η^2^ = 0.751/*p* < 0.001), followed by storage time (η^2^ = 0.401/*p* < 0.001). Storage conditions and all other combinations besides material*storage time (η2 = 0.043/*p* < 0.001) provided moderate impact (0.253 ≤ η^2^ ≤ 0.077/*p* < 0.001). The materials showed significantly (Anova *p* < 0.001) different baseline gloss values (Fig. 3).

LuxaPrint Ortho Plus showed gloss units (GU) between 62 (4 weeks water) and 114 (baseline). Significant differences were found after 2 weeks in coffee, mouth rinse, red wine, or air and after 4 weeks in water (Anova *p* ≤ 0.005, Bonferroni *p* ≤ 0.039). Optimill crystal clear provided significant general differences (Anova *p* ≤ 0.004) and individual differences (*p* ≤ 0.024) after 2 weeks storage in coffee, water, or red wine and after 2 h in mouth rinse and air. Gloss varied between 75 GU (4 weeks coffee) and 117 GU (baseline). KeySplint Soft showed gloss values between 12 GU (24 h red wine) and 51 GU (baseline). Significant general differences (Anova *p* ≤ 0.0.01) and individual differences (*p* ≤ 0.031) after 24 h storage for coffee, mouth rinse, red wine storage, or air and 2 weeks in water could be determined. Palapress vario provided significant general differences (Anova *p* ≤ 0.026) and individual differences (*p* ≤ 0.023) after 2 weeks storage in red wine and 4 weeks in coffee or mouth rinse. Gloss values between 64 GU (4 weeks red wine) and 86 GU (baseline) were found. Erkodur provided gloss results between 52 GU (4 weeks coffee) and 116 GU (baseline). Erkodur showed significant general differences (Anova *p* ≤ 0.001) and individual differences (*p* ≤ 0.039) after 24 h storage in coffee or water and 2 weeks in mouth rinse, red wine, or air. Gloss for Splint Flex varied between 59 GU (2 weeks coffee) and 92 GU (24 h red wine). Only general significant differences (Anova *p* = 0.007) were found for coffee storage. V-Print splint provided no significant general differences (Anova *p* ≥ 0.435) nor individual differences (*p* < 0.001) only in air. Changes were significant after 2 weeks for storage for coffee and in water (*p* ≤ 0.019) and after 4 weeks (*p* ≤ 0.006). Gloss varied between 78 GU (4 weeks red wine) and 101 GU (baseline) were found.

Gloss changes due to toothbrush abrasion: Highest gloss was found for Optimill crystal clear (144 baseline) and lowest for Erkodur (12 after toothbrush abrasion) with significant differences between the groups (Anova *p* ≤ 0.001). Toothbrush treatment significantly (*p* ≤ 0.024) influenced gloss for all materials, but KeySplint Soft (*p* = 0.061). Clearest influence of toothbrush abrasion was found for Erkodur with a gloss change of 85 units. Smallest influence was provided by KeySplint Soft with a gloss change of 9 units.

### Roughness

**Fig. 4:**
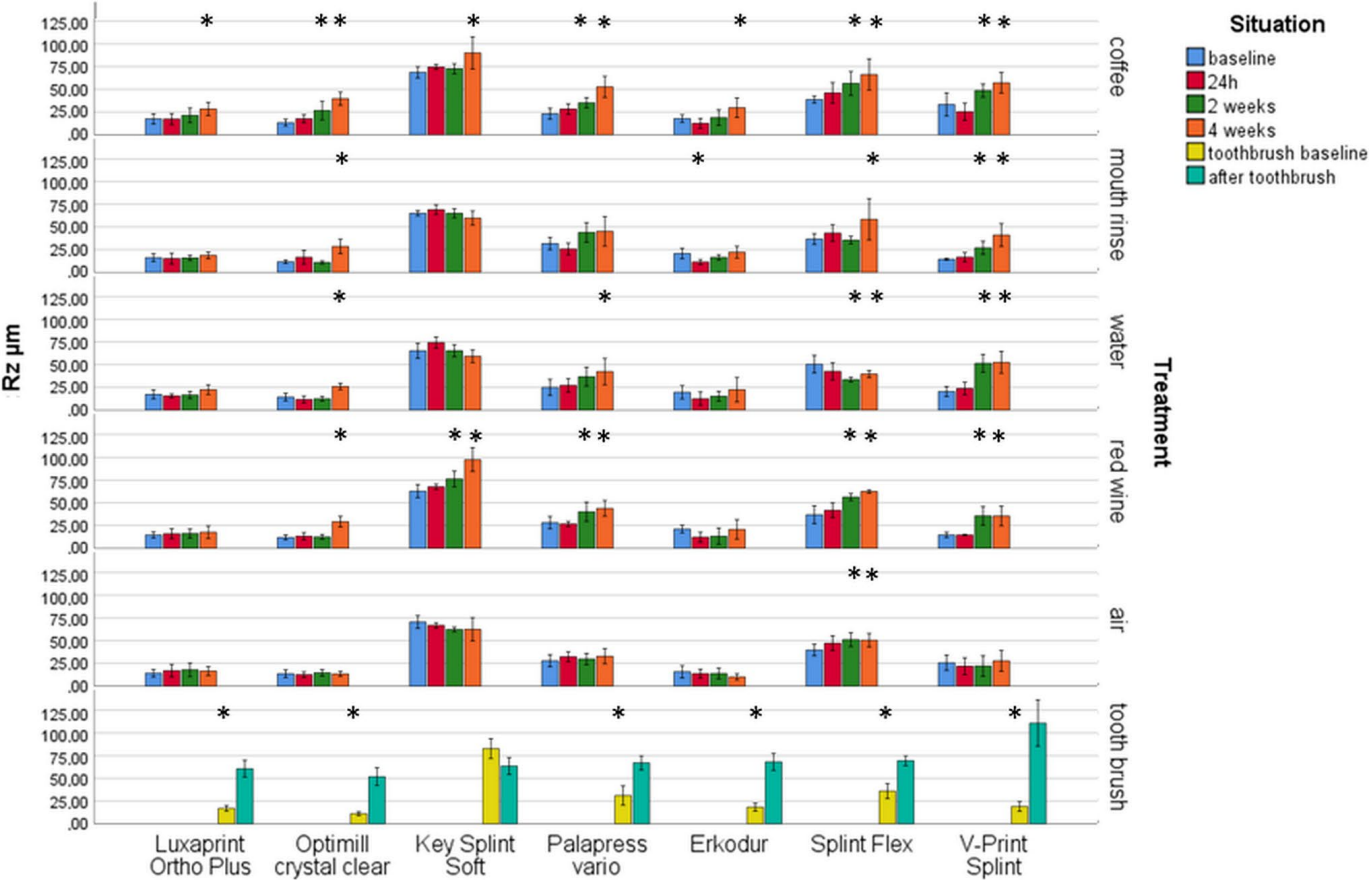
Surface roughness Rz after different aging/storage treatments and storage times (mean and standard deviation, * significant differences to baseline measurement, *α* = 0.05)

Highest impact on Ra and Rz (η^2^ ≥ 0.801/*p* < 0.001) presented the material, followed by storage time (η^2^ ≥ 0.416/*p* < 0.001) and their combinations (η^2^ ≥ 0.317/*p* < 0.001). The other combinations showed lower, but significant impact (0.072 ≤ η^2^ ≤ 0.271/*p* < 0.001). Both Rz and Ra provided significant (*p* ≤ 0.001) differences for the materials already in the baseline measurement (Fig. 4).

Significant Rz differences were found for LuxaPrint Ortho Plus after 4 weeks in coffee (*p* = 0.006). Optimill crystal clear provided significant differences (*p* = 0.001) after two 2 weeks coffee and 4 weeks mouth rinse, water, or red wine (*p* ≤ 0.001). KeySplint Soft showed significant (*p* = 0.000) differences after 4 weeks coffee and after 2 weeks red wine (*p* = 0.009). Significant (*p* ≤ 0.006) differences were found for Palapress vario after 2 weeks coffee or red wine and after 4 weeks water (*p* = 0.005). Erkodur provided significant (*p* = 0.008) differences after 4 weeks coffee and 24 h mouth rinse (*P* = 0.001). For Splint Flex, significant (*p* ≤ 0.021) differences could be determined after 2 weeks coffee, water, red wine, or air and after weeks mouth rinse (*p* = 0.004). V-Print splint showed significant (*p* ≤ 0.013) differences after 2 weeks coffee, mouth rinse, water, or red wine.

LuxaPrint Ortho Plus provided significant (*p* ≤ 0.035) Ra changes after 2 weeks coffee or mouth rinse. Significant changes were found for Optimill crystal clear after 24 h coffee (*p* = 0.014) and 2 weeks mouth rinse, water, or red wine (*p* ≤ 0.001). KeySplint Soft showed significant (*p* ≤ 0.046) changes after 2 weeks coffee, mouth rinse, or water and 24 h in red wine or air. Palapress vario provide significant (*p* ≤ 0.013) differences after 24 h in coffee, water, or red wine and 2 weeks mouth rinse (*p* = 0.020). For Erkodur, significant (*p* ≤ 0.018) changes were found after 24 h mouth rinse, water, or red wine and 4 weeks air (*p* = 0.048). Splint Flex provided significant (*p* ≤ 0.001) changes after 24 h water, red wine, or air and 2 weeks coffee. For V-Print splint, the changes were significant (*p* ≤ 0.007) after 24 h coffee, mouth rinse, water, or red wine.

Toothbrush treatment significantly influenced Rz (*p* ≤ 0.002) for all materials. Ra results were significantly (*p* ≤ 0.018) reduced for all materials but Optimill crystal clear (*p* = 0.327).

**Fig. 5:**
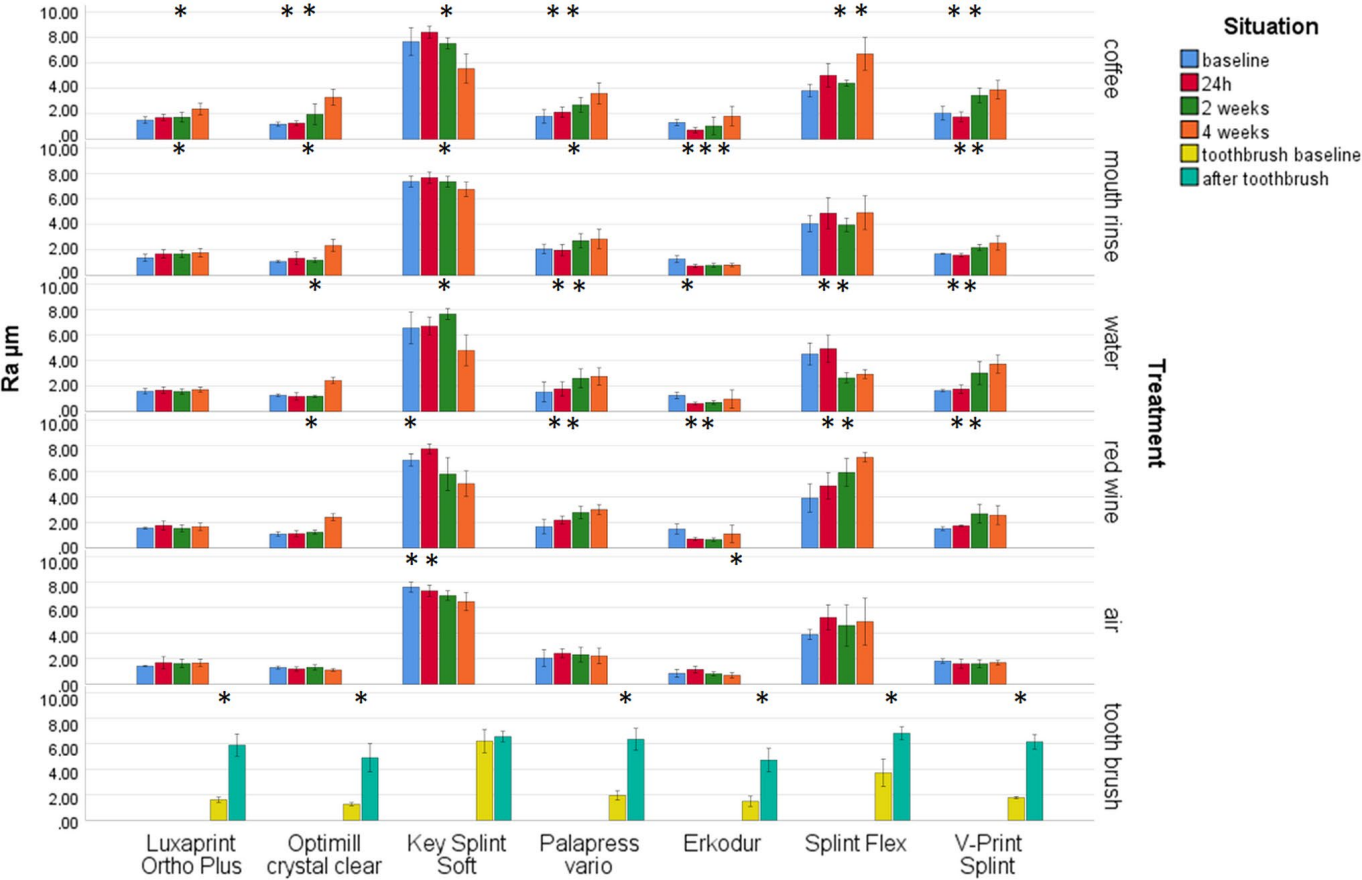
Mean surface roughness Ra after different aging/storage treatments and storage times (mean and standard deviation, * significant differences to baseline measurement, *α* = 0.05)

Significant correlations were found between Rz and Ra (Pearson 0.887/*p* ≤ 0.001) or Rz and ΔE (0.517/*p* ≤ 0.001) or Ra and ΔE (0.460/*p* ≤ 0.001). Significant negative correlations could be determined for gloss and Rz (− 0.714/*p* ≤ 0.001), Ra (− 0.712/*p* ≤ 0.001), and ΔE (− 0.558/*p* ≤ 0.001). *p* values of the Shapiro–Wilk normal distribution varied between 0.052 and 0.970 (color), 0.059 and 0.991 (gloss), 0.50 and 0.935 (roughness Ra), and 0.055 and 1.000 (roughness Rz) (Fig. 5).

## Discussion

The hypothesis of this in vitro study could be confirmed. Color, gloss, and surface roughness altered due to contact with staining solutions/toothbrush simulation and the changes were dependent on the individual material, the type, and duration of storage. There were no clear differences between the material groups. Influences on other properties such as hardness or polymerization [37–39], which were found for other printed materials, should be investigated in further tests and compared with the current data.

### Color

Different materials responded differently to the individual storage solutions. After 4 weeks at the latest, the ΔE values of all materials except LuxaPrint Ortho Plus reached or exceeded the acceptable ΔE value of 3.3 [24] in at least one of the storage media. The highest discoloration was obtained for specimens stored in coffee, followed by red wine, cleaning tablets, and demineralized water. Previous studies showing that coffee causes the most color changes in resins compared to other staining solutions could be confirmed [30, 40]*.* This was explained by the fact that coffee contains yellow colorants with different polarities. Yellow dyes could be absorbed into the organic phase through their compatibility with the polymer phase of the resin [30, 41]. Some materials might also show stronger discoloration as a result of a higher water absorption capacity [42]. The fact that the discoloration of most materials in air is much lower than in systems with moisture storage may confirm this assumption. The hypothesis that water is crucial in comparison to staining is also supported by the fact that the discoloration in water was comparable to storage in the other media—except coffee. However, since changes can also be observed for the soft printing systems during storage at air, incomplete polymerization [43, 44], and chemical reactivity could be reasons for the discoloration [23, 23, 23, 45, 46] of these materials. It is not only the resin composition, but also the conversion that seems important. Therefore, not only the fabrication but also the post-processing (cleaning, polymerization) may influence the color stability, as well as other properties such as the flexural strength. The results showed that the best color stability can be achieved with both milled and printed materials. The comparison with flexible soft materials indicated that they do not necessarily perform worse than conventional, firmer printing, thermoforming, or hand-cast materials. Significant correlations can be found between color change and roughness values, indicating that a decisive factor for color stability is a smooth surface and thus an excellent polish. The rougher surfaces were therefore probably more susceptible to discoloration [47–50]. Also, toothbrush abrasion demonstrated the influence of surface roughness on color behavior. After toothbrush simulation, all materials except KeySplint Soft achieved significantly higher color values. An explanation for this effect is the increasing surface roughness, influencing the superficial reflection [51].

### Gloss

Considering that good polishing resulted in gloss values between 70 and 80 GU [26–28], all the examined materials, except for KeySplint Soft, had good baseline gloss values. The reduced polishability may be due to the lower hardness of the material. For the materials Luxaprint, Optimill, and Erkodur, even excellent gloss values of over 100 could be achieved. Due to the variety of each material, it was difficult to standardize the surface of the test specimens under laboratory conditions [52]. Gloss values are inversely proportional to color changes: Gloss values of all investigated resins decreased by immersion in the test solutions and with increasing storage time. There was a moderate impact from the type of test solution, while no changes were observed when stored at air. The reason for the reduction of gloss due to storage in the test solutions could be an increase in the surface roughness and thus a larger exposed surface area or an effect of discoloration. More likely, however, is a loss of gloss caused by water absorption on the material [2, 53]. This is supported by the fact that the gloss values in humid ambient conditions differed only slightly despite the different storage liquids, and that storage in air had a more pronounced effect on the gloss values. Toothbrush abrasion resulted in a significant reduction in gloss for all materials. Significant negative correlations can be found for gloss, roughness, and color values. KeySplint Soft is an exception here and both Voco materials (Splint Flex, V-Print splint) also show less decrease of gloss. This might be attributed to identical monomer or filler components. The reduction in gloss after toothbrushing is in most cases accompanied by increased surface roughness. The abrasion of the resin matrix and loss of surface particles could have caused changes in the surface topography [28, 54]*.* Murakami et al. and Heintze et al. concluded that tooth brushing also leads to microscopic and macroscopic roughness. The result is a diffuse reflection of the incident light, accompanied by a reduction in gloss [55, 56]. It can be assumed that the composition of the toothpaste also has an influence on the loss of the surface gloss; e.g., with a more abrasive toothpaste, it is easier to create cavities or particles from the paste can be rubbed into the material surface [57].

### Roughness

The variety of materials studied had the greatest influence on roughness: both roughness values already showed significant differences for the materials in the initial measurement. Softer materials were rougher. One possible explanation could be that materials with lower surface hardness are more susceptible to scratches [27] and worse polishable with conventional means. After storage in the various test solutions, the materials can be divided into three groups: The soft materials (KeySplint Soft and Splint Flex) showed highest roughness, followed by Optimill crystal clear and Erkodur, as a third group with LuxaPrint Ortho Plus, Palapress vario, and V-Print splint achieved lowest roughness values.

The storage time also influenced the surface roughness. It can be assumed that water absorption reduces the hardness, which in turn increases the surface roughness [58, 59].

After toothbrush abrasion, the surface roughness increased significantly for all materials. An exception is the material KeySplint Soft, where the Rz-value even decreased after abrasion and the Ra-value only slightly increased. This is certainly due to the low hardness of the material. It is noticeable that the roughness values in this study are far above the threshold of clinical relevance (Ra < 0.2 µm) described earlier [60, 61]. Reference measurements with manual scanning prove that the values measured with a 3D laser-scanning microscope are higher by a power of ten. The different measurement setup and, for example, the different reflection of the specimen surface may be noticeable [62]. As the splint resins examined in the present study are typically transparent, surface reflections or absorption could have affected the measurements. Since the discs were measured at different storage times, the advantage that the surface remained undamaged by the optical measurement prevails. It can be assumed that the material hardness has an effect on roughness. Light-curing occlusal splint resins are expected to have comparable hardness as auto-polymerizing systems [63, 64], but hardness of 3D-printed occlusal splint materials is influenced by the print angle [17]. Martens hardness and indentation modulus depend on post-polymerization and are expected to decrease after water storage [39] [62].

The individual layers in transparent materials may affect neither the optical color nor gloss measurements, nor the roughness measurements. The present specimens were printed with a layer thickness of 50 µm in order to match surface quality and printing time [37, 65, 66]. Samples, which were printed with a lower (e.g., 25 µm) or adaptive layer thickness (inside 50 µm and outside 25 µm), might perform better, because an influence of the print parameters on the surface quality has been confirmed earlier.

The storage of the test specimens in various solutions and brushing with a toothbrush is accompanied by a change in color, gloss, and roughness. Discoloration and reduction of gloss usually leads to a loss of esthetic properties and thus limit the acceptance of the splint. Storage conditions such as pH in solution appear to be related to the hydrophilicity of the matrix and the chemical composition of the filler, which in turn affect sorption and solubility [67]. Greater surface roughness can cause bacteria and microorganisms to adhere more easily to the materials, leading to inflammation [60, 61]. If splints are to be used over a longer period of time, color- and gloss-resistant materials should therefore be preferred. A clinical consequence could be that occlusal splints are polished at regular intervals, which reduces the surface roughness [68, 69], removes extrinsic discoloration [70], and improves the gloss [68, 71]. In this in vitro study, the parameters were observed after 4 weeks of storage; however, it can be assumed that the values increase with increasing storage time [72, 73]. Photo-polymerization variables influence the structure and subsequent thermal response of dental resin matrices [74], and therefore, the degree of polymer polymerization or monomer release may be a key to the decrease in gloss and the increase in roughness and should be investigated in further studies. It is known that a combined heat and light-post-curing unit can improve the degree of conversion of 3D printed occlusal splints [75] and, e.g., hardness [38] or the in vitro performance [14, 19].

## Conclusion

Almost all the materials tested showed visible discoloration after only 4 weeks of storage, especially in coffee. Gloss values decrease as storage time is increased, and the type of test solution has a moderate effect. The initial surface quality and polishability are better with harder materials. Despite low hardness, the soft materials do not become rougher due to storage.

## Clinical consequence

An influence of the splint materials on the investigated parameters could not be proven: printed and even softer splints can also show good color, gloss, and roughness resistance after 4 week application. Color, gloss, and roughness correlate in some, but not all respects.
